# Frontoparietal Brain Network Plays a Crucial Role in Working Memory Capacity during Complex Cognitive Task

**DOI:** 10.1523/ENEURO.0394-23.2024

**Published:** 2024-08-07

**Authors:** Nikita Otstavnov, Carlos Nieto-Doval, Giulia Galli, Matteo Feurra

**Affiliations:** ^1^Centre for Cognition and Decision making, Institute for Cognitive Neuroscience, Higher School of Economics University, Moscow 101000, Russia; ^2^Department of Psychology, Kingston University, London KT1 2EE, United Kingdom

**Keywords:** frontoparietal network, high-definition transcranial direct current stimulation, operation span task, working memory, working memory capacity

## Abstract

Recent neurophysiological studies provide inconsistent results of frontoparietal network (FPN) stimulation for altering working memory (WM) capacity. This study aimed to boost WM capacity by manipulating the activity of the FPN via dual-site high–definition transcranial direct current stimulation. Forty-eight participants were randomly assigned to three stimulation groups, receiving either simultaneous anodal stimulation of the frontal and parietal areas (double stimulation), or stimulation of the frontal area only (single stimulation), or the placebo stimulation (sham) to frontal and parietal areas. After the stimulation, we used an operation span task to test memory accuracy, mathematical accuracy, time of calculation and memorizing, and recall response time across the three groups. The results revealed an enhancement of memory accuracy and a reduction of time of calculation in the double stimulation group compared with that in others. In addition, recall response time was significantly decreased in the double and single stimulation groups compared with that in sham. No differences in mathematical accuracy were observed. Our results confirm the pivotal role of the FPN in WM and suggest its functional dissociation, with the frontal component more implicated in the retrieval stage and the parietal component in the processing and retention stages.

## Significance Statement

Simultaneous frontoparietal dual-site transcranial direct current stimulation significantly enhanced performance and reduced response times in a complex working memory task compared with those in single-site frontal and sham groups.

## Introduction

Working memory (WM) is associated with the activity of frontal and parietal areas, which constitute the frontoparietal network (FPN). As FPN determines the quality of processing during verbal and visual tasks, it may reflect the central executive of WM (Rottschy et al., 2012) and determine an individual's WM capacity (Cole et al., 2012).

High-definition (HD) transcranial direct current stimulation (tDCS) is a promising technique for increasing WM capacity. Unlike the inconsistent effect of traditional tDCS (Hill et al., 2016; Mancuso et al., 2016) applied over frontal (F3) or parietal (P3) areas, there is a more consistent evidence of a positive effect of HD-tDCS over the left frontal (Ke et al., 2019) and parietal areas (Gözenman and Berryhill, 2016) during WM tasks. According to a meta-analysis by Müller et al. (2022), the application of HD-tDCS resulted in a trend of WM improvement in accuracy but not in response times (Müller et al., 2022).

Most of the studies investigated the effects of the stimulation to either the frontal or the parietal components of the FPN. Very few studies investigated the effect of a dual-site stimulation of both FPN components simultaneously to WM performance. To our knowledge, such an approach was only adopted in the study by Hill et al. (2018). In that study, the effects of dual-site HD-tDCS of the posterior (P3, PPC) and frontal lobes (F3, DLPFC) were systematically evaluated using *N*-back task and EEG (Hill et al., 2018). Despite the physiological changes in evoked responses, the authors found no difference in WM performance between stimulation groups. This could be explained by the nature of the WM tasks as *N*-back task mostly correlates with WM processing and involves additional cognitive functions (Hotton et al., 2018). Complex WM tasks could be more suitable to examine behavioral effects and dissect the role of different network nodes for WM (Bayliss et al., 2003).

Based on the idea that both components of FPN are involved in WM functioning, we assume that their simultaneous stimulation would maximize the effects of HD-tDCS on WM capacity. To test this assumption, we applied simultaneous anodal HD stimulation to the DLPFC and PPC, single stimulation of the left DLPFC, and sham stimulation of the left DLPFC and left PPC to three separate groups of participants before they performed a complex WM task. We hypothesized that in the group with double stimulation, we would observe a significant performance enhancement at the complex WM task (increased capacity and decreased response times). Moreover, we hypothesized that the effect of HD-tDCS would be more pronounced for harder trials compared with that for easier ones.

## Materials and Methods

### Participants

We performed an a priori power analysis in G*Power to compute the required sample size for general linear analysis of variance (ANOVA) by the given alpha, power, and effect size (Faul et al., 2007). The power calculation indicated that 45 participants were required to detect an effect of moderate size (Hedges’ *g* = 0.28 according to Müller et al., 2022) with alpha = 0.05 and 1 − beta = 0.95 for the interaction between stimulation groups and difficulty levels.

We recruited 48 right-handed individuals (25 females; mean age, 25.2 ± 2.8 years). Inclusion criteria were not having a history of neurological disorders, no CNS-active medical drugs at least 1 month before the experiment, normal or corrected-to-normal vision, and having at least 9 years of education (correspond to finished secondary education). We considered only participants of age 20–35 years because at this age the WM capacity is relatively at its peak and stable (Ferguson et al., 2021).

The study was a single blind. Participants were randomly allocated to three groups. Participants in the double stimulation group (*n* = 17) received anodal stimulation simultaneously over the left DLPFC and the left PPC, participants in the single stimulation group (*n* = 16) received the stimulation over the left DLPFC only, and participants in the sham group (*n* = 15) received sham stimulation. Before the experiment, all participants were screened via questionnaires to check for no contraindications to tDCS (adapted from Rossi et al., 2009) and to collect demographic data about gender, educational level, and age. All participants were acquainted with possible stimulation side effects and signed informed consent. After the experiment participants fulfilled the questionnaire about stimulation side effects and applied memory strategies. The study protocol was designed in compliance with the Declaration of Helsinki and approved by the local Ethical Review Board of Higher School of Economics (Russia, Moscow).

Before statistical analyses, we used the methodology of Conway et al. (2005) to identify participants who performed poorly in the operation span task (OSPAN) due to lack of attention and excluded participants with overall mathematical accuracy of <80%. Using this criterion, one female participant from the double stimulation group was excluded from the analyses.

### tDCS

**Figure 1.**
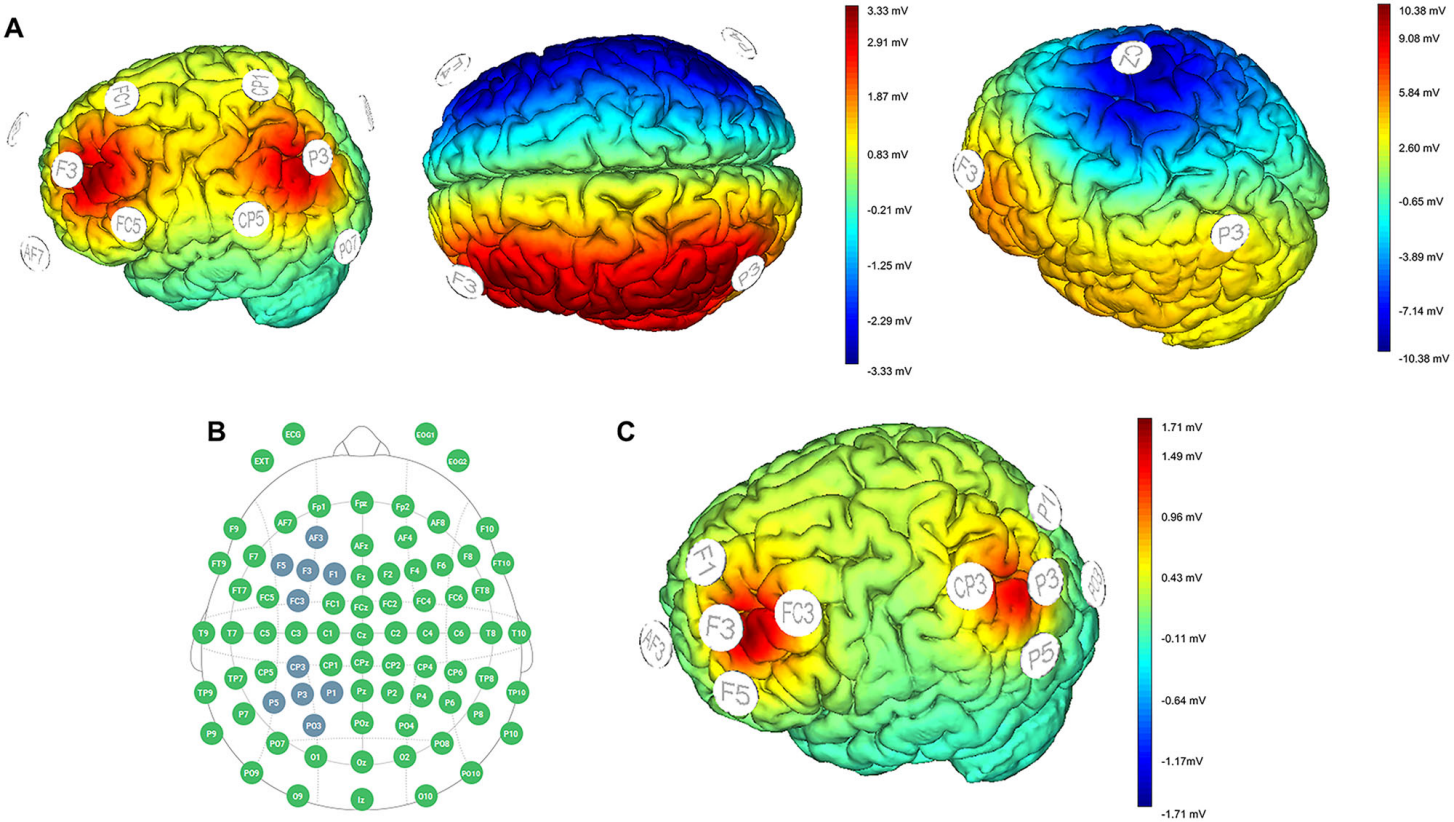
Simulation of current spread in the brain gray matter conducted with the NIC2 software. ***A***, Current spread for conventional and wide HD montages. ***B***, Electrode location according to 10/20 EEG system in the final HD montage. Central electrodes are anodal; peripheral electrodes are cathodal. ***C***, Current spread in the final HD montage, which was used in the study.

HD-tDCS was applied by a battery-driven Starstim32 stimulator (Neuroelectrics) via round 3.14 cm^2^ electrodes. All electrodes were placed using a 4 × 1 montage directly into a 64-channel EEG cap, where the central electrode upon the region of interest was anodal and the peripheral four electrodes were cathodal (Fig. 1). To target the left DLPFC, we placed the central electrode over F3 of the international 10–20 EEG system. To target the PPC, the central electrode was placed over P3. The montage was the same for all participants, except that in the dual-site group, the electrodes on both sites were active, whereas in the single stimulation group, only electrodes over F3 were active. The electrode montage was chosen based on the computational models of cortical electric fields created in the software NIC2 (v2.0.11.6, Neuroelectrics Instrument Controller). Different simulations with conventional tDCS montages showed that the simultaneous dual-site stimulation could not only affect the target brain regions but also spread to nearby regions, which could potentially bias the results (Lang et al., 2005). Therefore, the only montage that allowed influencing F3 and P3 focally without side effects was the HD montage (Fig. 1).

In the double and single stimulation groups, the stimulation was delivered for 15 min with an intensity of 1 mA for each active electrode (current density of 0.32 mA/cm^2^) and 0.25 mA per return electrodes. The ramping up and down stimulation period lasted for 30 s in total. The impedance level was controlled by using conducting gel. Impedances were kept below 10 kOm during the stimulation. In the sham group, the current switched off 15 s after the onset of the stimulation and switched back on 15 s before the end of the session. This induced a minor somatosensory sensation that successfully mimicked the stimulation: in the post-tDCS questionnaire, only one participant indicated correctly that they were in the sham group. OSPAN started within 5 min from the stimulation offset. In total, the behavioral part of the experiment lasted not >45 min, which was in the range of induced HD-stimulation effect (To et al., 2016).

In order to assess any possible side effect of the stimulation, we asked participants to fill out a post-tDCS questionnaire (adapted from Fertonani et al., 2010), where they had to indicate a range of somatosensory sensation felt during the stimulation (itching, pain, burning, heating, pinching, taste of iron/metal, fatigue) and whether they thought they received real or placebo stimulation.

### Task

**Figure 2.**
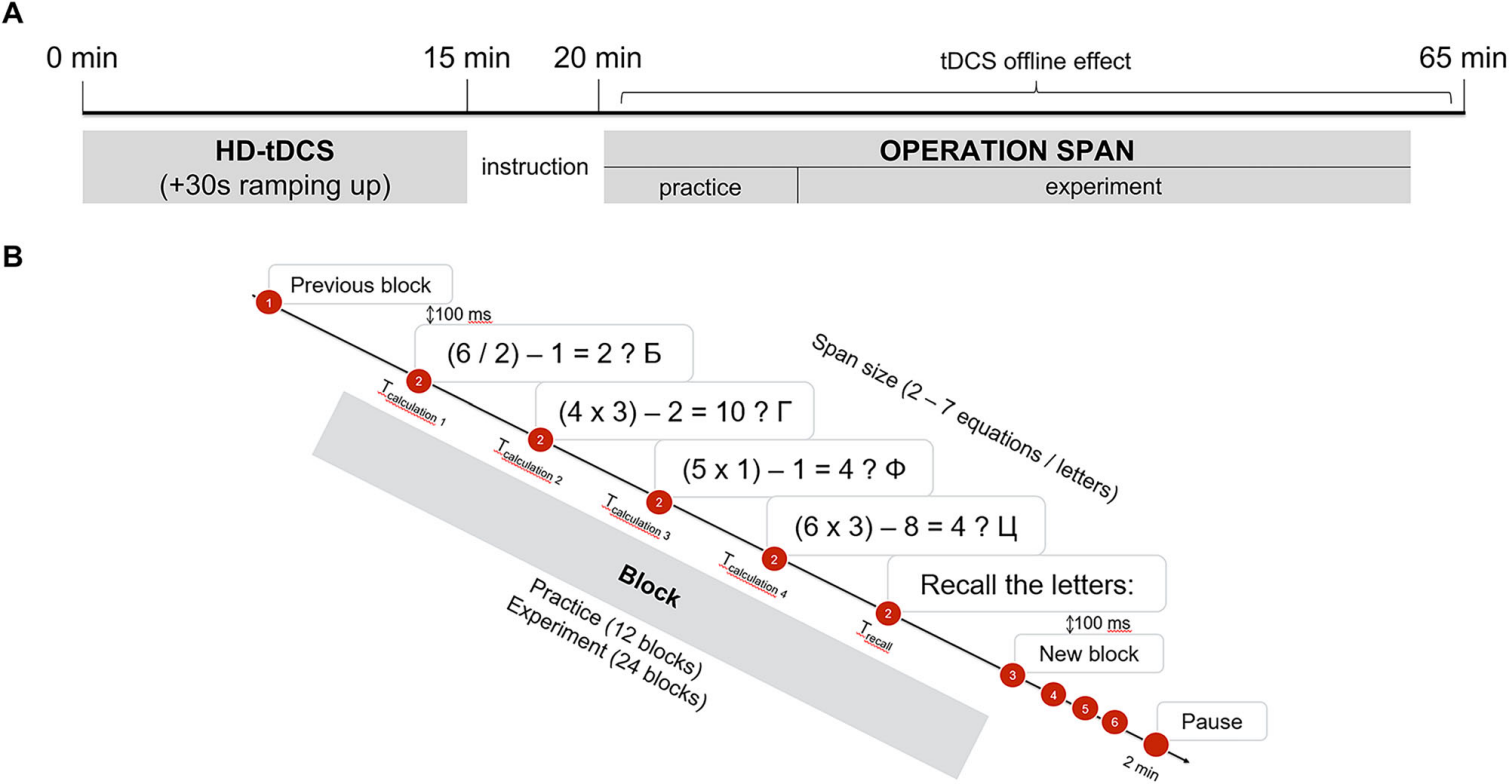
Overview of the experimental protocol. ***A***, Participants firstly received a stimulation and later perform a cognitive task. ***B***, Overview of the OSPAN. Participants were required to verify mathematical equations while simultaneously memorizing a varying set of letters. Before the experimental part, participants were asked to fulfill questionnaires to check for no contraindications to tDCS and to collect sociodemographic data. After the experimental part, participants were asked to fulfill questionnaires about stimulation side effects and applied memory strategies (Extended Data Fig. 2-1).

In a soundproof room, participants sat at ∼50 cm from a 27 in electron monitor with a 1,920 × 1,080 resolution and a 144 Hz refreshing rate. Figure 2 shows the design of the experimental protocol. We used an OSPAN to measure domain-general WM capacity. According to several studies, the OSPAN has good reliability (*α* coefficients of internal consistency, 0.8) and test-to-test correlations of 0.7–0.8 for several months (Kane et al., 2004; Conway et al., 2005).

10.1523/ENEURO.0394-23.2024.f2-1 Figure 2-1 Translated versions of questionnaires about tDCS contraindications, stimulation side effects and applied memory strategies. Participants fulfilled the contraindication questionnaire along with the consent form signing before the HD-tDCS setup. The TES side effects and memory strategies questionnaires were delivered to participant immediately after they finished the Operation Span task. Download Figure 2-1, DOCX file.

In each trial, participants performed two tasks simultaneously. They had to verify the solution of a mathematical equation (e.g., “(5 × 1) + 1 = 12? B”) while memorizing a letter (Turner and Engle, 1989). This allows testing both the storage and the processing functions of WM. The time to verify equations during the actual experiment was calculated individually for each subject based on the practice response times and adapting the formula of Wahlheim et al. (2019): *T*_mean_ + 2 * *T*_sd_ (Wahlheim et al., 2019). In the practice part, the time to verify equations was not limited, but to keep participants attentive to the task participants, they were told that they only had 6 s to provide a response. After equations were verified, participants were asked to type the memorized letters (retrieval). All responses were given with a keyboard. We used Cyrillic letters to present and recall stimuli, as all respondents were native Russian speakers. To avoid the effect of letter rehearsal and attention distraction, participants had to speak aloud the letters and equations.

The experiment consisted of 12 blocks of practice followed by 24 blocks of actual experiment. Task difficulty varied from two trials to seven trials in a block (Span Size 2, 3, 4, 5, 6, 7) in order to test HD-tDCS effect on the optimal (3–5) and boundary load (low, 2; high, 6 and 7; Cowan, 2001). During practice, participants performed each set size of trials twice, which allowed them to familiarize with the task structure and to get used to speaking letters and equations aloud. During the experimental part, participants performed each set size four times. In total, there were 24 trials with pseudorandomized difficulty, split into four sections with six trials in each to avoid fatigue effects. Participants were allowed to have 2 min break in-between blocks. The paradigm was designed and run in PsychoPy (v2021.1.4) in OS Windows 10. The average time for the practice part was 11.2 ± 1.9 min and for the experimental part 17.5 ± 3.9 min.

In order to control whether the results of the experiment are not caused by different memory strategies applied by participants (usage of any mnemonics, merging letters into word, letter articulation, imagination of a scene), we asked them to fill out a memory questionnaire.

### Data analysis

We used a between-subject design to avoid practice effects accompanying OSPAN (Klein and Fiss, 1999). We conducted mixed-model ANOVA with stimulation type (Type) as a between-subject factor, which was our primary interest, and difficulty levels (Load) as within-subject factor to examine the effects of load. Low Load included Span Sizes 2 and 3; medium, Span Sizes 4 and 5; and high, Span Sizes 6 and 7.

ANOVA was applied for the following dependent variables: memory accuracy, mathematical accuracy, recall response times, and time of correct calculations. We used partial credit scoring to assess memory accuracy, indexed by the mean ratio of correctly recalled letters per block regardless of their position in the set (Conway et al., 2005). Mathematical accuracy was assessed as the block's mean ratio of correctly verified mathematical equations. Recall response time was assessed from the onset of the retrieval window until the subject pressed the “enter” button and proceeded with the subsequent trial. The time of calculation was counted from the onset of each trial until the keypress, indicating the end of equation verification. As letters and equations were processed at the same time, the time of calculation is the same as the time of memorizing the letters. All measurements were averaged across the block.

The data were checked for assumptions of ANOVA about common variance and sphericity using Levene's and Mauchly's tests (Kozak and Piepho, 2018), respectively (Extended Data Table 1-1). If the assumptions of sphericity was violated, the Huynh–Feldt correction was applied (Howell, 2013). The assumption of normality (checked by the Shapiro–Wilk test) was violated per se, due to not-normal distribution for all experimental metrics. We proceed with ANOVA because it is proven to handle such non-normalities (Latinus et al., 2010; Gore et al., 2016; Tremblay et al., 2023). Significant interactions were followed up with Bonferroni’s post hoc analyses (Table 1, Kozak and Piepho, 2018). The level of significance was set at 0.05. Data analysis was performed in SPSS v26.0.0.1 (International Business Machines).

**Table 1.** Statistical table

Data structure	Type of test	Power (95% C.I.)
Memory accuracy (beta distribution)		
• Double vs single	Mixed-model ANOVA, Bonferroni’s test	CI: 1.712, 10.260
• Sham vs single		CI: −2.924, 5.765
• Double vs sham		CI: 0.221, 8.910
Mathematical accuracy (beta distribution)		
Double vs single	Mixed-model ANOVA, Bonferroni’s test	CI: −2.316, 1.836
Sham vs single		CI: −3.674, 0.547
Double vs sham		CI: −0.787, 3.434
Time of memorizing and calculation (gamma distribution)		
Double vs single	Mixed-model ANOVA, Bonferroni’s test	CI: −1.468, −0.544
Sham vs single		CI: −0.338, 0.586
Double vs sham		CI: −1.336, −0.428
Recall response time (gamma distribution)		
Double vs single	Mixed-model ANOVA, Bonferroni’s test	CI: −0.960, 1.710
Sham vs single		CI: 0.877, 3.591
Double vs sham		CI: −0.502, 3.216

C.I. indicates confidence interval. Detailed information about assumption testing of mixed-model ANOVA are presented in Extended Data Table 1-1.

10.1523/ENEURO.0394-23.2024.t1-1. Table 1-1. Testing the data on ANOVA assumptions of sphericity and homogeneity. Download Table 1-1., DOCX file.

Furthermore, we examined whether external factors (sociodemographic parameters, memory strategies) could influence the results. We controlled an equitable distribution of the educational level and gender (categorical data) across three groups by the *χ*^2^ test. Additionally, we employed a one-way ANOVA to assess whether experimental groups were different in age (continuous data). To investigate whether participants from different groups employed distinct memory strategies during the test, we conducted a *χ*^2^ test. Finally, we conducted a nonparametric Kruskal–Wallis test for the results of the post-tDCS questionnaire (ordinal data) to find potential differences among the three groups.

### Data accessibility

The PsychoPy paradigm of OSPAN described in the paper is freely available online at https://github.com/Nikita13021995/Fronto-parietal-network-stimulation. The behavioral data are available upon the reasonable request.

## Results

We compared the performance between three stimulation groups. The three groups did not differ in education level (*χ*^2^_(4)_ = 4.455; *p* = 0.348) and gender (*χ*^2^_(2)_ = 0.912; *p* = 0.634) as evidenced by the *χ*^2^ test (Extended Data Tables 1-2, 1-3) and in age (*F*_(2,44)_ = 2.022; *p* = 0.144), as evidenced by one-way ANOVA.

### Time of calculation

**Figure 3.**
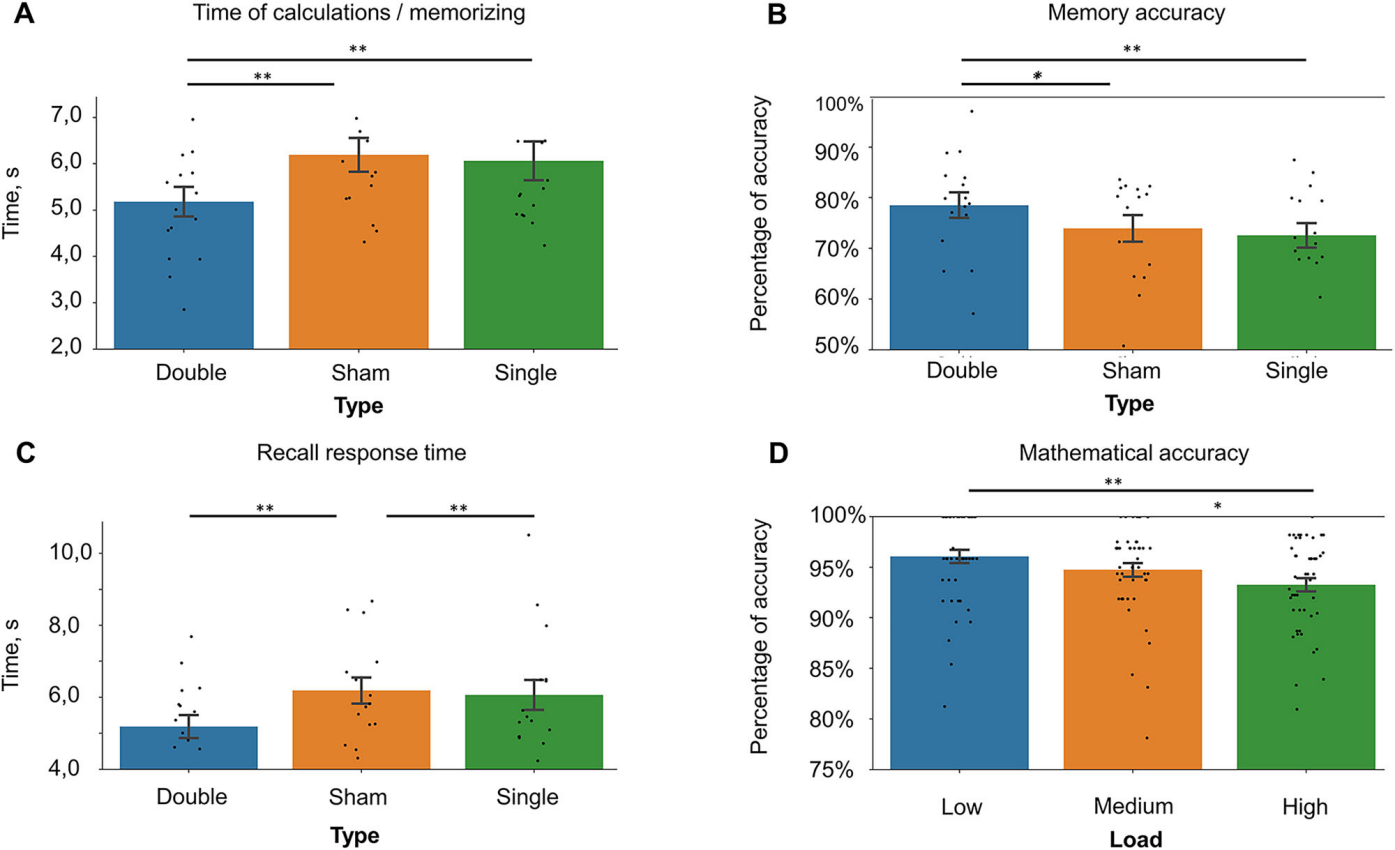
Change of operation span metrics concerning the stimulation protocol. Black dots indicate averaged individual performance. Mixed-model ANOVA was applied to analyze the group differences. Error bars represent standard error; *p < 0.05; ***p < 0.01. ***A***, Time of memorizing and calculation among stimulation types. ***B***, Memory accuracy among stimulation types. ***C***, Recall response time among stimulation types. ***D***, Mathematical accuracy for *N* = 47 in each group among difficulty levels.

Results for the time of calculation are shown in Table 2 and Figure 3*A*. The assumption of sphericity was violated. The mixed-model ANOVA revealed a significant main effect of Type (*F*_(2,373)_ = 16.601; *p* = 0.0001; partial *η*^2^ = 0.082). Post hoc comparisons revealed that the double stimulation group was significantly faster than the single and sham groups (double: sham, mean difference, −1.01 ± 0.19; *p* = 0.0001; double: single, mean difference, −0.88 ± 0.19; *p* = 0.0001; single: sham, mean difference, 0.12 ± 0.19; *p* = 0.796). The interaction between Type and Load was not significant (*F*_(4,746)_ = 1.607; *p* = 0.171; partial *η*^2^ = 0.009).

**Table 2.** Mean estimation of results for different types of stimulation (for trials averaged across load)

Group	Double (*N* = 128)		Single (*N* = 128)		Sham (*N* = 120)	
Metric	Mean value	Standard error	Mean value	Standard error	Mean value	Standard error
Memory accuracy	78.535%	1.257%	72.549%	1.257%	73.969%	1.298%
Mathematical accuracy	95.045%	0.610%	95.285%	0.610%	93.722%	0.630%
Time of calculation/memorizing	5.183	0.134	6.065	0.134	6.189	0.138
Time of recall	8.420	0.392	8.045	0.392	10.279	0.405

Detailed information about data distribution across groups is presented in Extended Data Table 2-1 (educational level), Table 2-2 (gender), Table 2-3 (guess about the group), and Table 2-4 (usage of mnemonics strategies).

10.1523/ENEURO.0394-23.2024.t2-1. Table 2-1. Contingency matrix for the distribution of education level categories across three stimulation groups. Download Table 2-1., DOCX file.

10.1523/ENEURO.0394-23.2024.t2-2. Table 2-2. Contingency matrix for the distribution of gender across three stimulation groups. Download Table 2-2., DOCX file.

10.1523/ENEURO.0394-23.2024.t2-3. Table 2-3. Contingency matrix for the distribution of participants who correctly guessed their group assignment. Download Table 2-3., DOCX file.

10.1523/ENEURO.0394-23.2024.t2-4. Table 2-4. Contingency matrixes for the distribution of participants who applied memory strategies such as Mnemonics, Merging letters into word, Articulation of the letters, Imagination of a scene. Download Table 2-4., DOCX file.

### Memory accuracy

Results for memory accuracy are shown in Table 2 and Figure 3*B*. The assumption of sphericity was violated. The mixed-model ANOVA revealed a significant influence of the between-subject factor Type (*F*_(2,373)_ = 6.173; *p* = 0.002; partial *η*^2^ = 0.032) and no interaction between Type and Load (*F*_(4,746)_ = 0.260; *p* = 0.903; partial *η*^2^ = 0.001). Post hoc comparisons revealed that the group with double stimulation performed significantly better in comparison with others (double: sham, mean difference, 4.566% ± 1.807%; *p* = 0.036; double: single, mean difference, 5.986% ± 1.777%; *p* = 0.003; single: sham, mean difference, −1.42% ± 1.807%; *p* = 0.99).

### Recall response time

Results for recall response times are shown in Table 2 and Figure 3*C*. The assumption of sphericity was violated. The mixed-model ANOVA revealed a significant main effect of Type (*F*_(2,373)_ = 8.906; *p* = 0.0001,;partial *η*^2^ = 0.046). Post hoc comparisons revealed that both stimulation groups were significantly faster than sham (double: sham, mean difference, −1.86 ± 0.56; *p* = 0.003; double: single, mean difference, 0.37 ± 0.56; *p* = 0.99; single: sham, mean difference, −2.23 ± 0.56; *p* = 0.0001). The interaction between Type and Load was not significant (*F*_(4,746)_ = 2.310; *p* = 0.065; partial *η*^2^ = 0.012).

### Mathematical accuracy

Results for mathematical accuracy are shown in Table 2 and Figure 3*D*. The assumption of sphericity was violated. The mixed-model ANOVA didn't reveal significant effect of Type (*F*_(2,373)_ = 1.823; *p* = 0.163; partial *η*^2^ = 0.010) nor interaction between Type and Load (*F*_(4,746)_ = 1.867; *p* = 0.117; partial *η*^2^ = 0.010).

### Load effect

The main effect of Load was significant for all metrics regardless the stimulation group (time of calculation, *F*_(2,746)_ = 14.827; *p* = 0.0001; partial *η*^2^ = 0.038; memory accuracy, *F*_(2,746)_ = 218.739; *p* = 0.0001; partial *η*^2^ = 0.370; recall response time, *F*_(2,746)_ = 181.173; *p* = 0.0001; partial *η*^2^ = 0.327; mathematical accuracy, *F*_(2,746)_ = 7.358; *p* = 0.001; partial *η*^2^ = 0.019). Post hoc comparisons are presented in Table 3.

**Table 3.** Post hoc comparison for load size (Bonferroni’s)

		Mean difference	Standard error	*p* value
Memory accuracy				
Low	Medium	14.240	1.449	0.0001**
Low	High	29.277	1.439	0.0001**
Medium	High	15.037	1.307	0.0001**
Time of calculation/memorizing				
Low	Medium	−0.303	0.056	0.0001**
Low	High	−0.175	0.059	0.01*
Medium	High	0.129	0.052	0.044*
Recall response time				
Low	Medium	−4.415	0.333	0.0001**
Low	High	−8.021	0.471	0.0001**
Medium	High	−3.606	0.450	0.0001**
Mathematical accuracy				
Low	Medium	1.332	0.883	0.396
Low	High	3.111	0.824	0.001**
Medium	High	1.779	0.727	0.045*

* *p* < 0.05.

** *p* < 0.01.

### Analysis of the questionnaires

The Kruskal–Wallis test indicated no differences among the three groups for all potential side effects of TES: itching (*H*_(2)_ = 3.091; *p* = 0.213), pain (*H*_(2)_ = 0.995; *p* = 0.608), burning (*H*_(2)_ = 0.250; *p* = 0.883), heating (*H*_(2)_ = 1.469; *p* = 0.480), pinching (*H*_(2)_ = 0.959; *p* = 0.619), taste of iron (*H*_(2)_ = 0.000; *p* = 1.0), and fatigue (*H*_(2)_ = 1.604; *p* = 0.448). No statistical differences were found in participants’ perception regarding the group assignment: *χ*^2^_(6)_ = 2.422; *p* = 0.877 (Extended Data Table 1-3). Only one participant correctly indicated that he was in the sham group.

We found no differences among the three groups in the memory strategies during the OSPAN (Extended Data Table 1-4): general usage of mnemonics (*χ*^2^_(8)_ = 11.206; *p* = 0.190), chunking (*χ*^2^_(6)_ = 6.157; *p* = 0.406), verbalization during the experiment (*χ*^2^_(4)_ = 4.058; *p* = 0.398), and mental visualization (*χ*^2^_(6)_ = 12.161; *p* = 0.058). Thus, we can conclude that the differences in OSPAN performance were driven by stimulation effect.

## Discussion

To our knowledge, this is the first investigation into the effect of simultaneous excitatory stimulation of two unilateral brain areas assessed with a complex WM span task. Many previous studies investigated the effect of anodal stimulation on either frontal (F3) or parietal (P3) areas separately (Fregni et al., 2005; Andrews et al., 2011; Heimrath et al., 2012; Pope et al., 2015; Heinen et al., 2016). In our experiment, participants performed a complex WM task after receiving double stimulation, single stimulation, or sham over the left frontal and parietal areas. Intriguingly, we observed a general positive effect of the dual-site stimulation protocol on WM performance, which indicates the importance of synchronized neuroenhancement of both FPN components. This aligns with our initial hypothesis that FPN components work together as two separate and highly connected neuronal units.

The dual-site stimulation increased memory accuracy and reduced time of calculation in comparison with other groups. Single and sham stimulation groups were not different from one another. The absence of effects of the single frontal stimulation suggests that the parietal area is crucial for stimulus storage and effective information processing. This assumption corresponds to the idea of the parietal involvement in attentional mechanisms of WM (Jonides et al., 1998) or WM search processes (Linden, 2007) and is in line with several studies (Duncan et al., 2003; Ezzyat and Olson, 2008).

Interestingly, for the recall phase of the WM task, both stimulation groups showed reduced recall response times compared with sham. This highlights the critical involvement of the frontal area in retrieval from WM. Since there was no memory accuracy enhancement in the single group, we can assume that stimulation of the frontal area boosts the speed of information retrieval from WM but not the quality (accuracy) of such retrieval. This assumption confirms the involvement of the frontal lobe in selecting relevant information from several alternatives (Thompson-Schill et al., 2002) and is in line with several studies (Rypma and D’Esposito, 1999). The DLPFC is usually associated with general executive functioning (Wager and Smith, 2003; D’Esposito et al., 2000) or with attention focusing on task-relevant information (Faraco et al., 2011), thus controlling the effective encoding or retrieval strategies and boosting the recall response time.

Altogether, these results highlight the differential role of FPN nodes in complex tasks. The interconnection between frontal and parietal areas is essential to perform dual tasks (Gulbinaite et al., 2014; Violante et al., 2017); however, the frontal area is more crucial for the speed of retrieval, while the parietal area is crucial for efficient processing (D’Esposito et al., 1999; Linden, 2007).

The results generally align with the study by Hill and collaborators, which compared dual-site stimulation of the FPN with single-site stimulation during the *N*-back task (Hill et al., 2018). Although Hill et al. did not find visible behavioral improvements in performance in the double stimulation group, they reported increased theta and gamma power following the dual-site stimulation. Frontoparietal theta power is usually connected with maintaining the information by supporting the long-range communication between the frontal and parietal areas (Albouy et al., 2022). Gamma power reflects active WM processing (Bonnefond and Jensen, 2015) and is related to successful WM encoding and retrieval (Graetz et al., 2019). In our experiment, we used an OSPAN, which combines short-term memory retention with the executive (interfering) task and thus can be a more sensitive measure of WM capacity. Altogether, double stimulation could boost the connection between separate frontal and parietal nodes in theta and gamma ranges via their coupling (Jones et al., 2020), thus strengthening the storage and processing functions of WM, which can be further examined by single parietal stimulation protocol. Nonetheless, such interpretations should be made with caution, as the results may be influenced by the stimulation parameters of the current study (electrode sites, electrode number, current density) or the experimental design (stimulus language, number of set size repetitions, number of practice trials) and may not be simply generalizable to other tDCS setups. These considerations should be addressed through future systematic evaluations.

The significant difference across Loads observed in our experiment for memory accuracy and time indicates that the experimental paradigm was well controlled. We constructed trials with varying difficulty to assess the effects of the stimulation on different WM load conditions. However, we did not observe an interaction between the stimulation group and Load, which contradicts our hypothesis of larger effects of the stimulation in harder trials.

Some limitations of our study need to be considered. As Hill and colleagues showed in their meta-analysis, the effects of tDCS are inconsistent across studies (Hill et al., 2016). Most of the studies with tDCS were conducted on a small sample size. Our sample was sufficiently powered to detect both an effect of the stimulation and the interaction between stimulation and difficulty level. However, due to the absence of studies with complex WM spans, the parameters for conducting the power analysis were taken from studies with other WM tasks, leading to some approximation. The fact that for this study we applied a between-group design is the second limitation. From the one side, such design allows avoiding practice effect, which can detrimentally affect the results of OSPAN (Klein and Fiss, 1999). On the other side, it is a limitation, because we cannot rule out that the observed differences are not due to differences from a baseline performance. Future studies should control tDCS effects by including a baseline performance measurement where it is possible. Another limitation is related to the possibility that the results could be influenced by the spread of electrical currents along the head surface rather than by the brain stimulation. However, this possibility is generally negligible with HD-tDCS, because nonadjacent electrodes are used to overcome leakage and electrical spread (Nikolin et al., 2019), which was confirmed by the simulations. Finally, in our experiment, we did not check whether the effect of HD-tDCS was specific to WM using a separate cognitive task because it prolonged the experimental session beyond the duration of peak neuroplasticity effect after stimulation (Kuo et al., 2013). Nevertheless, we can refer to mathematical operation as a distinct cognitive task, which can partially serve as the control task (Turner and Engle, 1989). According to our results, the accuracy of mathematical task was not affected by the stimulation.

In conclusion, this study provided novel insights into the effects of concomitant stimulation of the parietal and frontal nodes of the FPN using HD-tDCS. The results emphasize the differential contributions of the FPN nodes and underscore the significance of boosting network synchronization for WM performance during complex WM tasks. Overall, this study highlights the novelty and effectiveness of concomitant stimulation of the FPN in enhancing WM performance. The observed improvements provide valuable insights for potential clinical applications in treating disorders characterized by decreased WM capacity.

## Synthesis

Reviewing Editor: James Howard, Brandeis University Decisions are customarily a result of the Reviewing Editor and the peer reviewers coming together and discussing their recommendations until a consensus is reached. When revisions are invited, a fact-based synthesis statement explaining their decision and outlining what is needed to prepare a revision will be listed below. The following reviewer(s) agreed to reveal their identity: NONE.

Your manuscript resubmission has been seen by one additional reviewer. The reviewer has a few concerns that must be addressed before publication can be considered. These concerns are summarized as follows:

1. Please acknowledge either on the Methods or in the Discussion section that the electrode sites used for stimulation may differ from other published studies, i.e. there is not a consensus on the number and exact location of electrodes necessary to test the hypothesis of the current study.

2. Please include a justification for the total number of practice trials (12) and experimental trials (24). Were other trial numbers considered and/or tested? Is this similar to other studies in the OSPAN literature? Do the authors speculate that these numbers would be different if the sample included non-Russian native speakers?

3. There is concern that the authors' rejection of an alternative explanation of their findings regarding altered inhibition does not logically flow from the cited literature on altered inhibition. It is suggested that the authors attempt to argue this point using more closely related literature (i.e., not reducing the literature on inhibition to the two cited studies), or to remove this paragraph entirely.
