# Supplementary material for: Frontoparietal Brain Network Plays a Crucial Role in Working Memory Capacity during Complex Cognitive Task
Source: eNeuro. 2024 Aug 7;11(8):ENEURO.0394-23.2024. doi: 10.1523/ENEURO.0394-23.2024 (PMC11315429; doi:10.1523/ENEURO.0394-23.2024)
Supplement: Figure 2-1 — Translated versions of questionnaires about tDCS contraindications, stimulation side effects and applied memory strategies. Participants fulfilled the contraindication questionnaire along with the consent form signing before the HD-tDCS setup. The TES side effects and memory strategies questionnaires were delivered to participant immediately after they finished the Operation Span task. Download Figure 2-1, DOCX file. [file eneuro-11-ENEURO.0394-23.2024-s006.docx]

**Extended Data Figure 2-1.**

Screening questionnaire before TES/TMS procedures
(based on updated questionnaire by Rossi, 2009)
The questionnaire was adapted for Russian-speaking participants

1. Do you have epilepsy or have you ever had a convulsion or a seizure?
2. Have you ever had a fainting spell or syncope? If yes, please describe on which occasion(s)?
3. Have you ever had a head trauma that was diagnosed as a concussion or was associated with loss of consciousness?
4. Do you have any hearing problems or ringing in your ears?
5. Are you pregnant or is there any chance that you might be? Are you a woman while breastfeeding?
6. Do you have metal in the brain, skull or elsewhere in your body (e.g., splinters, fragments, clips, etc.)? If so, specify the type of metal.
7. Do you have cochlear implants?
8. Do you have an implanted neurostimulator (e.g., DBS, epidural/subdural, VNS)?
9. Do you have a cardiac pacemaker or intracardiac lines?
10. Do you have a medication infusion device?
11. Are you taking any medications or taken during the latest half of a year? (Please list)
12. Did you ever have a surgical procedure to your spinal cord?
13. Do you have spinal or ventricular derivations?
14. Do any of your relatives have an epilepsy?
15. Do you suffer from constant headaches? Were you diagnosed with migraines?
16. Were you diagnosed with any neurological diseases?
17. Did you ever undergo TES or TMS in the past?
18. Did you ever undergo EEG in the past? If so, were there any problems.
19. Did you ever undergo MRI in the past? If so, were there any problems.
20. Did you consume alcohol (or any substances) during the day before the experiment?
21. Did you drink more coffee during last day than usual? Did you drink coffee later than 4 hours before the experiment?
22. How much sleep did you get two last nights (hours)? Did you sleep less two last nights less than you usually do?

Screening questionnaire after TES/TMS procedures of possible side effects.
(adapted from Fertonani et al., 2010)

1. Did you experience any discomfort or discomfort during stimulation?
2. Please answer possible questions, require different needs, and apply the degree of discomfort using the scale:
   1. No - I did not feel this sensation (0)
   2. Slightly - I felt the specified sensation slightly (1)
   3. Moderate - I felt the specified sensation (2)
   4. Noticeable - I felt the indicated sensation quite strongly (3)
   5. Strong - I strongly felt the specified sensation (4)

- Itching
- Pain
- Burning
- Heating
- Pinching
- Taste of iron/metal
- Fatigue

1. How long did the discomfort last?

- It ended quickly
- Ended in the middle of the block
- Continued until the end of the block

1. How strongly did these feelings affect the performance of the task?

- Not affected at all
- Slightly
- Medium
- Strongly
- Very strongly

1. Were the sensations on the head or in some other place?

- On the head
- Elsewhere

1. Do you think you got real stimulation, or a placebo?

- Real
- Placebo

Memory questionnaire after the experiment.

1. Did you used any specific strategy to memorize the letters?
   ________________________________________________________
   ________________________________________________________
2. While memorizing did you tried to merge letters into words or verbal combinations?
   yes

- yes
- no
- other: _________________________________________________

1. While memorizing did you articulate the letters constantly?

- yes
- no
- other: _________________________________________________

1. While memorizing did you imagine letters in particular vivid scene?

- yes
- no
- other: _________________________________________________
