# Supplementary material for: Frontoparietal Brain Network Plays a Crucial Role in Working Memory Capacity during Complex Cognitive Task
Source: eNeuro. 2024 Aug 7;11(8):ENEURO.0394-23.2024. doi: 10.1523/ENEURO.0394-23.2024 (PMC11315429; doi:10.1523/ENEURO.0394-23.2024)
Supplement: Table 1-1. — Testing the data on ANOVA assumptions of sphericity and homogeneity. Download Table 1-1., DOCX file. [file eneuro-11-ENEURO.0394-23.2024-s001.docx]

Extended Data Table 1-1.

| Assumption | Span | Memory | Mathematical accuracy | Time of calculation / memorization | Time of recall |
| --- | --- | --- | --- | --- | --- |
| Sphericity (Mauchly's Test) | | χ^2^_(2)_ = 6.213  p = 0.045 * | χ^2^_(2)_ = 18.327  p < 0.001 * | χ^2^_(2)_ = 6.469  p = 0.039 * | χ^2^_(2)_ = 59.457  p < 0.001 * |
| Homogeneity of varience (Levene’s median test) | Low load | F_(2, 373)_ = 1.933  p = 0.146  F_(2, 373)_ = 2.087  p = 0.125  F_(2, 373)_ = 0.253  p = 0.777 | F_(2, 373)_ = 0.410  p = 0.664  F_(2, 373)_ = 2.063  p = 0.128  F_(2, 373)_ = 3.757  p = 0.024 ** | F_(2, 373)_ = 1.119  p = 0.328  F_(2, 373)_ = 1.059  p = 0.348  F_(2, 373)_ = 0.777  p = 0.461 | F_(2, 373)_ = 1.132  p = 0.324  F_(2, 373)_ = 5.090  p = 0.007 *  F_(2, 373)_ = 1.396  p = 0.249 |
|  | Medium load |  |  |  |  |
|  | High load |  |  |  |  |
|  | Between-group non-parametric test |  | F_(2, 376)_ = 5.964,  P = 0.051  (high load) |  | F_(2, 376)_ = 16.986,  p < 0.001 ***  (medium load) |

** Significant Mauchly's test. For the cases of violation of sphericity the Huynh-Feldt correction was used.*

*** Significant test. For the heterogenic data additional Kruskal-Wallis test was performed and reported in brackets.*

**** Significant main effect of the between-group factor*
