# Supplementary material for: Frontoparietal Brain Network Plays a Crucial Role in Working Memory Capacity during Complex Cognitive Task
Source: eNeuro. 2024 Aug 7;11(8):ENEURO.0394-23.2024. doi: 10.1523/ENEURO.0394-23.2024 (PMC11315429; doi:10.1523/ENEURO.0394-23.2024)
Supplement: Table 2-1. — Contingency matrix for the distribution of education level categories across three stimulation groups. Download Table 2-1., DOCX file. [file eneuro-11-ENEURO.0394-23.2024-s002.docx]

Extended Data Table 2-1.

| Stimulation group | Unfinished  Bachelor degree | Bachelor degree | Master degree | Overall |
| --- | --- | --- | --- | --- |
| Sham | 5 | 7 | 4 | 16 |
| Double | 4 | 6 | 5 | 15 |
| Single | 4 | 11 | 1 | 16 |
| Overall | 13 | 24 | 10 | 47 |
