# Supplementary material for: Frontoparietal Brain Network Plays a Crucial Role in Working Memory Capacity during Complex Cognitive Task
Source: eNeuro. 2024 Aug 7;11(8):ENEURO.0394-23.2024. doi: 10.1523/ENEURO.0394-23.2024 (PMC11315429; doi:10.1523/ENEURO.0394-23.2024)
Supplement: Table 2-2. — Contingency matrix for the distribution of gender across three stimulation groups. Download Table 2-2., DOCX file. [file eneuro-11-ENEURO.0394-23.2024-s003.docx]

Extended Data Table 2-2.

| Stimulation group | Female | Male | Overall |
| --- | --- | --- | --- |
| Sham | 9 | 6 | 15 |
| Double | 7 | 9 | 16 |
| Single | 9 | 7 | 16 |
| Overall | 25 | 22 | 47 |
