# Supplementary material for: Frontoparietal Brain Network Plays a Crucial Role in Working Memory Capacity during Complex Cognitive Task
Source: eNeuro. 2024 Aug 7;11(8):ENEURO.0394-23.2024. doi: 10.1523/ENEURO.0394-23.2024 (PMC11315429; doi:10.1523/ENEURO.0394-23.2024)
Supplement: Table 2-3. — Contingency matrix for the distribution of participants who correctly guessed their group assignment. Download Table 2-3., DOCX file. [file eneuro-11-ENEURO.0394-23.2024-s004.docx]

Extended Data Table 2-3.

|  | Were assigned to experimental group | Were assigned to sham group | Overall |
| --- | --- | --- | --- |
| Thought to belong to the experimental group | 23 | 14 | 37 |
| Thought to belong to the sham group | 9 | 1 | 10 |
| Overall | 32 | 15 | 47 |
