# Supplementary material for: Frontoparietal Brain Network Plays a Crucial Role in Working Memory Capacity during Complex Cognitive Task
Source: eNeuro. 2024 Aug 7;11(8):ENEURO.0394-23.2024. doi: 10.1523/ENEURO.0394-23.2024 (PMC11315429; doi:10.1523/ENEURO.0394-23.2024)
Supplement: Table 2-4. — Contingency matrixes for the distribution of participants who applied memory strategies such as Mnemonics, Merging letters into word, Articulation of the letters, Imagination of a scene. Download Table 2-4., DOCX file. [file eneuro-11-ENEURO.0394-23.2024-s005.docx]

Extended Data Table 2-4.

| Mnemonics | Participants, who used this memory strategy | Participants, who used this memory strategy sometimes | Participants, who didn’t use this memory strategy | Overall |
| --- | --- | --- | --- | --- |
| Sham | 10 | 0 | 5 | 15 |
| Double | 6 | 3 | 7 | 16 |
| Single | 8 | 2 | 6 | 16 |
| Overall | 24 | 5 | 18 | 47 |

| Merging letters into word | Participants, who used this memory strategy | Participants, who used this memory strategy sometimes | Participants, who didn’t use this memory strategy | Overall |
| --- | --- | --- | --- | --- |
| Sham | 9 | 1 | 5 | 15 |
| Double | 10 | 0 | 6 | 16 |
| Single | 9 | 2 | 5 | 16 |
| Overall | 28 | 3 | 16 | 47 |

| Articulation of the letters | Participants, who used this memory strategy | Participants, who used this memory strategy sometimes | Participants, who didn’t use this memory strategy | Overall |
| --- | --- | --- | --- | --- |
| Sham | 8 | 7 | 0 | 15 |
| Double | 8 | 7 | 1 | 16 |
| Single | 12 | 4 | 0 | 16 |
| Overall | 28 | 18 | 1 | 47 |

| Imagination of a scene | Participants, who used this memory strategy | Participants, who used this memory strategy sometimes | Participants, who didn’t use this memory strategy | Overall |
| --- | --- | --- | --- | --- |
| Sham | 0 | 13 | 2 | 15 |
| Double | 3 | 13 | 0 | 16 |
| Single | 0 | 15 | 1 | 16 |
| Overall | 3 | 41 | 3 | 47 |
